# A Rare Case of Small Bowel Intussusception in an Elderly: A Case Report and Literature Review

**DOI:** 10.7759/cureus.44204

**Published:** 2023-08-27

**Authors:** Rohan Raj, Maya Ann Francis, Deepa Treesa Francis, Parvinder Kaur, Rupert Chima, Niyaz A Jamil

**Affiliations:** 1 Internal Medicine, Nalanda Medical College and Hospital, Patna, IND; 2 Internal Medicine, Windsor University School of Medicine - St Kitts and Nevis, Cayon, KNA; 3 Internal Medicine, Crimean State Medical University, Simferopol, UKR; 4 Internal Medicine, Cardiocare Multispeciality Hospital Abuja, Abuja, NGA; 5 Surgery, Windsor University School of Medicine, Cayon, KNA; 6 Surgery, Combined Military Hospital, Nowshera, PAK

**Keywords:** ostomy, resection, case report, carcinoma, bowel obstruction, intussusception

## Abstract

Intussusception, a rare cause of bowel obstruction in adults, is even less common in the elderly population. Unlike pediatric cases, adult intussusception is primarily associated with pathologic diseases acting as lead points, often requiring surgical intervention. We present a case of an 84-year-old male with a medical history significant for multiple comorbidities, who was diagnosed with a large segment jejunojejunal intussusception resulting in small bowel obstruction. Surgical management was recommended, and an exploratory laparotomy with bowel resection was performed, including the excision of the leading point. This case highlights the challenges in diagnosing adult intussusception and the importance of surgical intervention due to the high incidence of associated pathologic diseases.

## Introduction

Intussusception is the telescoping or invagination of the proximal gastrointestinal tract into an adjacent section, requiring surgical correction to avoid bowel ischemia or perforation. Intussusception is an uncommon cause of adult intestinal obstructions, even rare in older people. All adult intussusception cases should be managed with surgical therapy, with the timing dependent on the patient's clinical condition. Adult patients, as opposed to children, need an exploratory laparotomy and resection, which includes the removal of the intussusception's focal site and any nonviable intestine found during the surgical inspection [1]. Only 1% to 5% of intestinal obstructions in adults are due to adult intussusception. In comparison to its pediatric equivalent, this illness shows a number of distinctions [1]. While air-contrast enemas and pneumatic or hydrostatic reduction are successful in treating intussusception in around 80% of cases involving adolescents, more than 90% of adult cases are associated with an underlying medical disease that serves as a trigger. The pathogenic elements often found during surgical operations include strictures, polyps, Meckel's diverticulum, carcinomas, colonic diverticula, or benign neoplasms [2,3]. Adults seldom undergo radiologic decompression as a preoperative procedure since there is a greatly increased risk of concomitant malignancy, which is found in around 65% [4]. Here we present a case of an 84-year-old male with small bowel obstruction secondary to intussusception, who was surgically managed.

## Case presentation

We present a case of an 84-year-old male with a significant past medical history of chronic kidney disease (CKD), type II diabetes mellitus, hypertension, hyperlipidemia, and benign prostatic hyperplasia (BPH) who was brought to the Emergency Department following a mechanical fall and altered mental status. He was found to be unresponsive with hypoglycemia (blood glucose level of 45 mg/dL). As a result, he was admitted for further evaluation. Initial head computed tomography (CT) scan results were unremarkable. Due to abdominal distension, a CT scan of the abdomen and pelvis was performed, indicating suspected intussusception due to dilated bowel loops, and bowel-within-bowel appearance.

**Figure 1:**
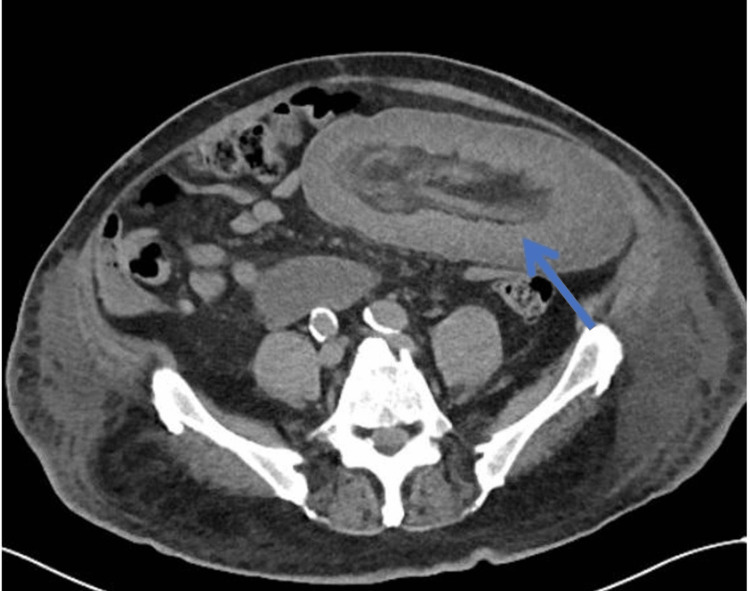
CT of the abdomen showing a large long segment likely jejunojejunal intussusception resulting in small bowel obstruction with edematous changes of the involved bowel loops (arrow).

On review of the systems, the patient denied abdominal pain and nausea. He experienced some nausea during the placement of a nasogastric tube on the day of evaluation. He reported normal bowel movements three days prior to presentation and continued to pass flatus. He denied shortness of breath, chest pain, headache, paresthesia, limb weakness, and any other symptoms in other systems. Upon examination, the patient appeared alert and oriented with no acute distress. On head, eyes, nose, and throat (HENT) evaluation, he exhibited normocephalic status, intact extraocular movements, and no scleral icterus. A nasogastric tube was placed with approximately 700 cc of bilious output. He showed no signs of respiratory distress and exhibited non-labored respiration on 3 L of oxygen. Cardiovascular examination revealed a normal heart rate and regular rhythm with no murmurs, gallops, or rubs. There were no signs of peripheral edema or jugular venous distension. Distal pulses were strong and equal in all limbs. His abdomen was soft, distended, non-tender, with no rebound or guarding, and non-peritonitic. On musculoskeletal examination, no gross deformity of extremities was observed. The skin was warm and dry. On neurologic evaluation, the patient was found to be awake, alert, and oriented to person, place, and time. The psychiatric assessment showed him to be cooperative, with an appropriate mood, affect, and thought. Throughout his hospitalization, he exhibited normal lactate levels and no signs of leukocytosis. However, a repeat CT scan showed a large intussusception involving a long segment of the jejunum with an edematous appearance of the intussuscepted bowel within the mesenteric vessels (Figure 1). As a result, the surgical team was consulted, and surgical management was recommended.

**Figure 2:**
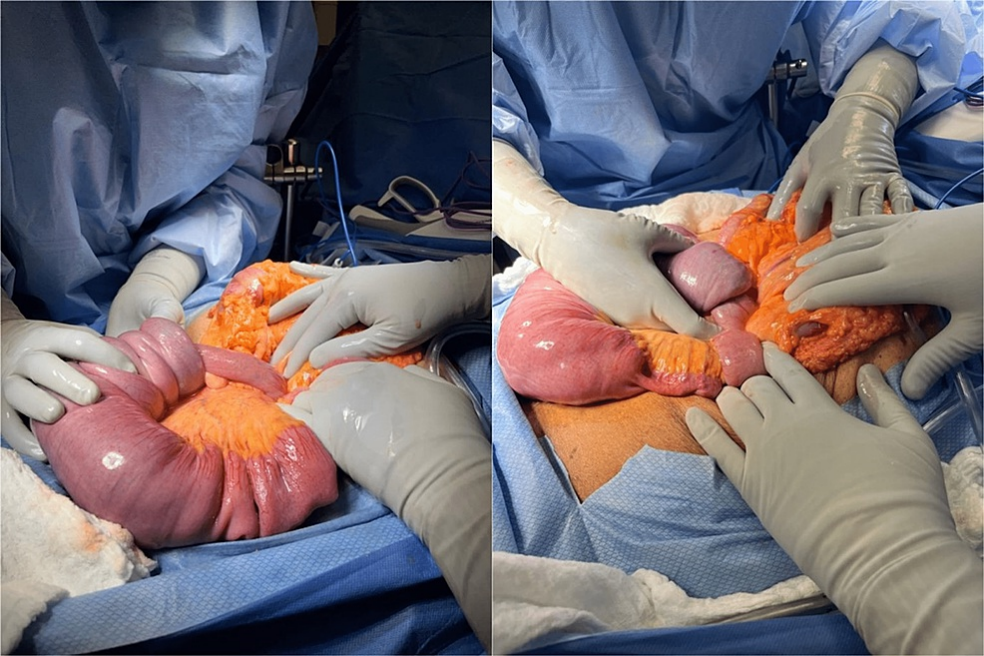
The intussuscepted bowel.

An assessment plan was made that the patient requires surgical management and the anesthesia team was consulted for exploratory laparotomy with possible bowel resection with either ostomy or primary anastomosis owing to the diagnosis of intussusception with small bowel obstruction. Written and informed consent was obtained from the patient and his wife. Cardiology and anesthesia consultations were completed. The patient was kept nil per oral (NPO), and a nasogastric tube was placed on low continuous wall suction. Antibiotics and heparin were administered as needed. A midline laparotomy incision was made, and the intussuscepted bowel was identified and reduced (Figure 2). A polyp that served as a lead point was found on the proximal portion of the bowel, approximately 10-15 cm distal to the ligament of Treitz. Around 60 cm of the small intestine along with the polyp was transected, and the bowel was anastomosed using staplers. The mesentery was transected with high ligation, including palpable lymph nodes. The peritoneal defect was approximated with a figure-of-eight silk suture, and the mesenteric defect was fixed with a running Vicryl suture. A crotch stitch was placed, and hemostasis was achieved. The fascia was closed with interrupted 1-0 Vicryl sutures. The patient had no signs of ischemia, and the rest of the intestine appeared healthy. The skin was closed in reverse fashion.

The patient's postoperative plan involved returning to the intensive care unit for further care. The procedure was performed successfully; the patient responded well to the given treatment and was discharged with out-patient follow-up.

## Discussion

The term "intussusception" describes when one section of the intestine invades another. Intussusception is the most common reason for bowel obstruction in babies, although it is very uncommon in adults, especially in the elderly. Three different types of intussusception can occur: enteric, ileo-colic, and colo-colic [5]. Clinical signs of mechanically induced intestinal blockage include nausea, vomiting, and cramping in the abdomen. Adult intussusception must be diagnosed with a high degree of suspicion, a thorough review of medical history, and a thorough physical examination.

The heterogeneity in both clinical presentation and imaging results makes it difficult and hard to identify intussusception prior to surgery. While Reijnen et al. [6] noticed a somewhat higher preoperative diagnosis rate of 50%, Eisen et al. [7] reported a diagnostic rate of 40.7%. Plain abdominal X-rays are commonly used as the initial diagnostic method since obstructive symptoms frequently predominate the clinical picture. These pictures frequently show the symptoms of intestinal obstruction and may provide information about the location of the obstruction [8]. A barium enema examination may be helpful in detecting intussusception, exhibiting a typical "cup-shaped" filling defect [9,10]. A characteristic "stacked coin" look could also be seen in the upper gastrointestinal contrast series.

For both adults and children, ultrasound is recognized as a useful diagnostic tool for detecting intussusception [11,12]. The existence of a "target" sign on the transverse view and the development of the "pseudo-kidney" sign on the longitudinal view are examples of classic imaging features. Abdominal CT has emerged as the most sensitive radiological method for verifying intussusception, with reported diagnostic accuracies ranging from 58% to 100% [13-15]. A "target"-shaped soft tissue mass with a layering effect is a prominent feature that can be noticed on a CT scan. Another distinguishing characteristic is the presence of mesenteric arteries within the intestinal lumen [16].

Adults may appear with acute, subacute, or chronic nonspecific presentations of their symptoms. The variety of symptoms makes it difficult to make an early diagnosis, which frequently forces doctors to wait until the patient is in the operating room. The majority of surgeons agree that adult intussusception requires surgical intervention because of the high prevalence of anatomical abnormalities and the possibility of cancer. However, there is still disagreement on how to manipulate the intussusceptible intestine during reduction and how much intestinal resection is necessary [16]. Regarding the laparoscopic treatment of adult intussusception caused by benign and malignant diseases of the small and large intestines, various studies have been published [17-20]. Depending on the overall health of the patient and the availability of surgeons with appropriate laparoscopic experience, laparoscopy has been utilized successfully in a few circumstances. Adult patients are not frequently advised to employ preoperative reduction procedures utilizing barium or air as a regular course of treatment, in contrast to pediatric patients, whose intussusception is typically primary and benign [6,16,17].

## Conclusions

Adult intussusception is an uncommon cause of bowel obstruction typically linked to underlying pathologic diseases, such as strictures, polyps, Meckel's diverticulum, carcinomas, colonic diverticulum, or benign neoplasms. Preoperative radiologic decompression is not recommended in adults due to the high incidence of concomitant cancer. A case report of an 84-year-old male with multiple comorbidities showed a large segment of jejunojejunal intussusception causing small bowel obstruction. Surgical management, including exploratory laparotomy and bowel resection, was performed, and the leading point of the intussusception was excised. Early diagnosis is crucial, especially given the higher prevalence of malignancy as a lead point in adult cases. Further research and collaborative efforts are needed to improve understanding and management of this uncommon condition in adults.
